# Research prioritisation on prevention and management of preterm birth in low and middle-income countries (LMICs) with a special focus on Bangladesh using the Child Health and Nutrition Research Initiative (CHNRI) method

**DOI:** 10.7189/jogh.13.07004

**Published:** 2023-09-01

**Authors:** Shumona Sharmin Salam, Shafiqul Ameen, Julie Balen, Quamrun Nahar, Sabrina Jabeen, Anisuddin Ahmed, Bronwen Gillespie, Lawrence Chauke, Abdul Mannan, Mahbubul Hoque, Sanjoy Kumer Dey, Jahurul Islam, Sabina Ashrafee, Husam Md Shah Alam, Ashfia Saberin, Palash Kumar Saha, Supriya Sarkar, Azizul Alim, Muhammad Shariful Islam, Clive Gray, Shams El Arifeen, Ahmed Ehsanur Rahman, Dilly OC Anumba

**Affiliations:** 1 The University of Sheffield, Sheffield, UK; 2 International Centre for Diarrhoeal Disease Research Bangladesh (icddr,b), Dhaka, Bangladesh; 3 Canterbury Christ Church University, Canterbury, UK; 4 University of the Witwatersrand, Johannesburg, South Africa; 5 Bangabandhu Sheikh Mujib Medical University (BSMMU), Dhaka, Bangladesh; 6 Dhaka Shishu (Children) Hospital, Dhaka, Bangladesh; 7 Directorate General of Health Services (DGHS), Government of Bangladesh, Ministry of Health and Family Welfare, Bangladesh; 8 Stellenbosch University, Stellenbosch, South Africa

## Abstract

**Background:**

Fifteen million babies are born preterm globally each year, with 81% occurring in low- and middle-income countries (LMICs). Preterm birth complications are the leading cause of newborn deaths and significantly impact health, quality of life, and costs of health services. Improving outcomes for newborns and their families requires prioritising research for developing practical, scalable solutions, especially in low-resource settings such as Bangladesh. We aimed to identify research priorities related to preventing and managing preterm birth in LMICs for 2021-2030, with a special focus on Bangladesh.

**Methods:**

We adopted the Child Health and Nutrition Research Initiative (CHNRI) method to set research priorities for preventing and managing preterm birth. Seventy-six experts submitted 490 research questions online, which we collated into 95 unique questions and sent for scoring to all experts. A hundred and nine experts scored the questions using five pre-selected criteria: answerability, effectiveness, deliverability, maximum potential for burden reduction, and effect on equity. We calculated weighted and unweighted research priority scores and average expert agreement to generate a list of top-ranked research questions for LMICs and Bangladesh.

**Results:**

Health systems and policy research dominated the top 20 identified priorities for LMICs, such as understanding and improving uptake of the facility and community-based Kangaroo Mother Care (KMC), promoting breastfeeding, improving referral and transport networks, evaluating the impact of the use of skilled attendants, quality improvement activities, and exploring barriers to antenatal steroid use. Several of the top 20 questions also focused on screening high-risk women or the general population of women, understanding the causes of preterm birth, or managing preterm babies with illnesses (jaundice, sepsis and retinopathy of prematurity). There was a high overlap between research priorities in LMICs and Bangladesh.

**Conclusions:**

This exercise, aimed at identifying priorities for preterm birth prevention and management research in LMICs, especially in Bangladesh, found research on improving the care of preterm babies to be more important in reducing the burden of preterm birth and accelerating the attainment of Sustainable Development Goal 3 target of newborn deaths, by 2030.

An estimated 11% of live births or 14.8 million babies are born preterm globally every year [1]. Preterm birth complications are the leading cause of death among newborns (n = 0.88 million (36.1%)) and under-five children (n = 0.94 million, (7.7%)), particularly in South Asia and East Asia and the Pacific region [2-4]. Additionally, the increasing rate of preterm birth in most countries and the insufficient rate of decline in preterm-related deaths ( ~ 1%) resulted in an increasing proportion of preterm-related deaths in newborns and under-five children [1,5], with proportionate mortality due to preterm birth complications increasing from 14.5% to 17.6% in the latter group between 2000 and 2019 [4]. If this trend continues, preterm birth complications will remain the leading cause of neonatal and under-five deaths even in 2030, at the end of the Sustainable Development Goals (SDGs) era [4,5].

Although high- and lower-resource countries do not differ drastically in rates of preterm birth, they do not share the resulting burden equally due to a large survival gap in preterm newborns between them and a substantially higher impact of preterm birth low- and middle-income countries (LMICs) [1,2,6]. LMICs also face challenges in coverage, quality, and equity of essential reproductive, maternal, newborn, and child health (RMNCH) interventions needed for the prevention and management of preterm births [7,8,9]. Simultaneously, other factors such as exposure to air pollution [10-12], low or advanced maternal age [13], poor maternal nutrition [14,15], and infections [16,17] are either higher or increasing among women in these settings. The problem has been exacerbated by the coronavirus 2019 (COVID-19) pandemic, which adversely affected patients, health care workers, and health systems in LMICs that had already struggled with various health challenges before the pandemic. Recent reviews indicated that COVID-19 may not only be associated with increased risks of preterm birth, pre-eclampsia (an indirect cause of preterm birth), and other adverse pregnancy outcomes [18], but the indirect effect of disruption in routine health care, including those for preterm birth and access to food, would result in between 253 500 (least severe) to 1 157 000 (most severe) additional child deaths [19].

Bangladesh, a LMIC in South Asia, witnessed high neonatal and child mortality declines during a two-decade period starting in the early 1990s. However, this progress has stalled since 2010, requiring a re-evaluation of the current strategic focus and interventions to avert deaths due to preventable causes such as birth asphyxia, pneumonia, and prematurity [20,21]. Bangladesh is one of the top five contributors to the global burden of preterm births and low birth weight (LBW) complications, which are estimated to be responsible for 15 000 deaths yearly [1,20]. Preterm and LBW complications account for approximately 13% of all child deaths and 19% of deaths among newborns in the country, making them the third and second leading causes of death in children and newborns, respectively [20,21]. Between 2014 and 2017, the proportion of deaths due to prematurity increased higher than for any other cause, by 1.7 times in newborns and 1.9 times in under-five children [20,21].

Advancing a research agenda for the prevention and management of preterm neonates across the continuum of care is critical to addressing the burden of preterm births in LMICs, and specifically, in Bangladesh [23,24]. Research to understand the causes, mechanisms, and risks before, during, and between pregnancies will help with the development and implementation of innovative strategies for preterm birth prevention [9,23-25]. However, implementation research is critical in increasing the uptake and scaling up of evidence-informed preterm birth prevention and care interventions, including those outlined in the Every Newborn Action Plan (ENAP) (e.g. kangaroo mother care (KMC), antenatal corticosteroid, special care newborn units, etc.) in ways that are practical and affordable [8,26]. In the context of increasing funding constraints, there is a need to prioritise and guide research efforts to achieve maximum impact on mitigating preterm births to attain the SDG target of reducing newborn and under-five child mortality by 2030.

Given that preterm birth complications will continue to be the leading cause of newborn and child mortality, stagnation in efforts to address this issue may hinder progress towards achieving the SDG-3 targets in Bangladesh and many low-resource settings. The NIHR Global Health Research Group on Preterm Birth Prevention and Management (PRIME), therefore, undertook a research priority-setting exercise using the Child Health and Nutrition Research Initiative (CHNRI) method [26]. Since the recognition of the “10/90 gap” in health research investments [27.28], several approaches have been employed over the past two decades to prioritise global health research needs across various settings, ranging from informal non-replicable consultation methods to the more comprehensive structured approaches [29,30]. Among the structured approaches that offer more transparency and replicability, the CHNRI method has been used repeatedly with over 100 applications and has become a often-applied approach for research priority setting [29,31]. This method, developed between 2005 and 2007, relies on the collective opinion of experts to systematically list and transparently score many competing research questions using predefined criteria [26]. Past research priority-setting exercises related to preterm birth, conducted by Bahl et al. [32] and George et al. [33] using the CHNRI method, offered important insight, but focused on the global level and improving progress towards attaining the Millennium Development Goal (MDG) 4. Here we report the research priorities needed for preventing and managing preterm birth for LMICs and Bangladesh in the post-MDG era and highlight considerations for successful implementation.

## METHODS

### Study design

**Figure 1:**
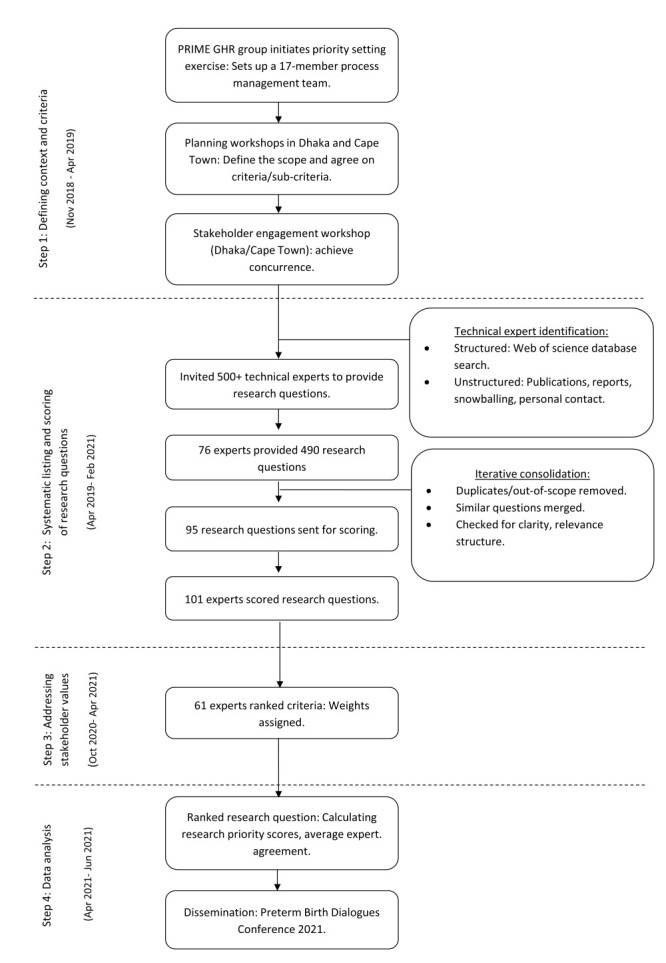
Steps in the CHNRI research priority setting process.

We adopted the CHNRI method [26] to set research priorities for preventing and managing preterm birth LMICs. The exercise involved four main steps to establish a list of priority research questions (**Figure 1**).

#### Step 1: Defining the context and criteria

We identified and established a process management team of 17 members from the PRIME collaborating institutions in Bangladesh, South Africa and the UK, entrusting them to coordinate the research priority-setting exercise. All team members were experienced in conducting maternal and newborn health research and included biomedical and laboratory scientists, health systems and public health researchers, clinicians, and social scientists. The team conducted planning workshops in Bangladesh and South Africa to define the scope and scoring criteria for the priority-setting exercise. Online workshops were later held to iteratively review and consolidate research questions and discuss the research prioritisation activity results.

The process management team discussed and specified the context of this research priority exercise in terms of space, population, and time; disease, disability, and death burden; and research domains. The goal of this phase was to identify research questions that have the potential to reduce the burden of premature birth and accelerate the progress towards achieving SDG Target 3 in LMICs, with a particular focus on Bangladesh as a high-burden country (**Table 1**). Following the CHNRI methodology, the team decided to encourage longer-term (up to 10 years) investments and included research questions from all four broad research domains (epidemiological research to describe the burden and determinants, health systems and policy research to improve the delivery of current interventions, research to improve existing interventions, and research to develop new interventions).

**Table 1:** Context for the research priority setting exercise

Geographical area	In LMICs and Bangladesh
**Time period**	SDG period (2021-2030)
**Target population**	Women of reproductive age (15-49 y); pregnant women; preterm/ LBW / SGA babies.
**Targeted disease burden**	PROM, premature birth, LBW, SGA, short- and long-term morbidity in preterm babies.
**Research domain**	Health policy and system research; research to improve existing interventions; development of new interventions; epidemiological research to understand burden and risk factors.

LMICs – low and middle-income countries, SDG – Sustainable Development Goals, LBW – low birthweight, SGA – small for gestational age, PROM – premature rupture of membrane, y – year

The process management team carried out a ranking exercise followed by a detailed discussion to identify four to five criteria for the priority-setting exercise. The team reviewed all the criteria identified by Rudan et al. [26] and scored them based on their relative importance in scoring research questions (range = 1-10). **Table 2** presents the top five criteria selected for the exercise. Since the agreed criteria were the same as the standard CHNRI criteria, the process management team also decided to retain the standard sub-criteria used to score research questions [26].

**Table 2:** List of selected criteria used to score research questions

Criteria	Explanation	Weights
Answerability	The research question can be ethically answered	1.01
Effectiveness	The research question will generate/improve truly effective health interventions	1.08
Deliverability	The intervention resulting from the research question will be deliverable	0.93
Maximum impact on burden	The research question has greater potential to reduce disease burden	1.08
Equity	The intervention resulting from the research will be accessible to vulnerable groups thus decreasing equity	0.90

#### Step 2: Systematic listing of research questions

We combined structured and unstructured approaches to identify global or international and local (Bangladesh) technical experts in the field of preterm birth research (**Box 1**). This included a bibliometric search of the Web of Science Core Collection database to identify the most scientifically productive researchers (with available contact details) in this field in the last 10 years, including those based in LMICs. Additionally, we enlisted experts from prior CHNRI priority-setting exercises related to preterm birth, authors of reviews on preterm birth, keynote speakers at preterm birth-related conferences, and other related researchers based on personal communication. We also encouraged the invited experts to share the survey links within their network. The local (Bangladesh) experts included members from the national newborn technical working committee, the ministry of health, obstetric, gynaecological, newborn, and paediatric professional bodies, development partners, service providers, and researchers working in maternal and newborn health with interest in preterm birth. We made efforts to invite experts from diverse disciplines (e.g. researchers, clinicians, policymakers, programme implementers) and countries (including LMICs) to participate in the survey.

Box 1 **Identification of technical experts in the field of preterm birth research**

− Listing from previous CHNRI exercises
−  GAPPS Expert Group et al. [33]
−  Bahl et al. [32]
−  Yoshida et al. [37]
− Listing from other publications
−  Born too Soon report
−  PRIME mapping review
−  Separation and Closeness Experiences in the Neonatal Environment (SCENE)
− Keynote speakers in Preterm Birth Dialogues Conference (South Africa)
− Author list of articles published on preterm birth published in the last 10 years found in Web of Science Core Collection database
− Personal Communication
− Snowballing

An online platform was developed and email invitations were sent to over 500 global technical experts to participate in the research priority-setting exercise. In the first online survey, we asked the technical experts to systematically list research questions on preventing and managing preterm birth across the four research domains according to the predefined context. The exercise was open to all research methodologies and study designs (e.g. observational, randomised trials, modelling, etc.). Participants could submit up to 10 priority research questions for each research domain based on their knowledge and expertise. Seventy-six technical experts (60 global and 16 from Bangladesh) submitted 490 research questions (an average of 6.4 research questions per person) between July and December 2019. Around two-thirds of the experts were public health and health systems researchers, while a half were involved in clinical services. Around 90% of the technical experts were experienced in working in LMICs (**Table 3**).

**Table 3:** Characteristics of technical experts participated in first and second online survey*

Characteristics	First online survey: Listing research questions (n = 76)	Second online survey: Scoring research questions (n = 109)	*P*-value†
**Area of expertise**			
Laboratory science research	9 (12)	8 (7)	0.244
Clinical science research	36 (47)	39 (36)	0.134
Public health and health systems research	49 (64)	69 (63)	0.889
Policy and programme implementation	30 (39)	45 (41)	0.785
Clinical services (direct health care)	38 (50)	45 (41)	0.226
**Experienced in working in LMIC**			
Yes	66 (87)	104 (95)	0.052
No	10 (13)	5 (5)	0.052
**Experienced in working in South Asia**			
Yes	41 (54)	78 (72)	0.012
No	35 (46)	31 (28)	0.012
**Experienced in working in Africa**			
Yes	44 (58)	35 (32)	0.000
No	32 (42)	74 (68)	0.000
**Location (international/local (Bangladesh)**			
Local (Bangladesh)	16 (21)	54 (50)	0.000
International	60 (79)	55 (50)	0.000
**Years of experience in working in LMIC, mean (SD)**	16 (12)	25 (10)	0.000
**Years of experience in working in MNH, mean (SD)**	23 (12)	26 (10)	0.066

SD – standard deviation, LMIC – low and middle-income country, MNH – Maternal and Newborn Health

*Values presented as n (%) unless otherwise specified.

†We used proportion test for frequencies and two sample independent *t* test for mean. *P* < 0.05 indicates statistical significance.

A few members of the process management team reviewed the research questions and collated them to a manageable number by research domains (**Table 1**) by removing questions outside of the scope of the exercise (e.g. not related to preterm birth), categorising and merging similar questions, and removing duplicates. We shared the ensuing list with the wider process management group, who reviewed the questions for relevance, clarity, and structure and reduced the list further to 95 unique questions through consultation. Most questions were from the epidemiological research domain (n = 38 (40%)) and health systems and policy research field (n = 33 (35%)), while a smaller number were on research to improve existing interventions (n = 15 (16%)) or to develop new interventions (n = 9 (9%)).

The technical experts who were invited to provide the research questions were invited again to score the final list of 95 research questions using a separate online platform (**Table 3**). In the second online survey, the technical experts were asked to score the final list of research questions against the criteria and sub-criteria selected in the first step. The following scores were assigned: “I agree” (1 point), “I neither agree nor disagree” (0.5 points), “I disagree” (0 points), and “Not well informed” (blank). We also randomly presented the research questions to technical experts to overcome bias due to scoring fatigue. We considered the responses valid if experts had scored at least one whole research question. One hundred and nine technical experts (55 international and 54 from Bangladesh) scored the questions based on five pre-selected criteria: answerability, effectiveness, deliverability, maximum potential for burden reduction, and effect on equity between October 2020 and April 2021.

#### Step 3: Addressing stakeholder values

During the PRIME project, we carried out extensive stakeholder engagement activities/workshops with policymakers, programme implementers, public health professionals, researchers, and health care service providers in Bangladesh. By introducing and ensuring their concurrence for the research priority-setting exercise, we wanted to increase the contextual relevance, acceptability, and eventual uptake of identified priorities. We also asked all experts to rank each predefined criterion based on their perceived relative importance using a five-point Likert scale (1 = least important, 5 = most important) (step four), which we then used to calculate weighted research priority scores. Seventy-six experts ranked the criteria; those for effectiveness (3.25) and potential for burden reduction (3.25) received the highest ranks, followed by answerability (3.02), deliverability (2.79), and equity (2.70).

#### Step 4: Data analysis and evaluation

We used a password-protected Microsoft Structured Query Language (SQL) Server 2008 R2 as the central database for maintaining the quality and safety of data. We set up validation rules (such as consistency checks, logical checks, and skip rules) to prevent inconsistencies and other errors during data entry.

We calculated intermediate, unweighted, and weighted research priority scores (RPS) for each of the five scoring criteria used to discriminate the 95 research questions for this exercise [26]. For each of the research questions, experts provided the following answers: 1 (agree), 0 (disagree), 0.5 (unsure) and blank (if experts were not informed enough to judge a research question). We calculated intermediate RPS by summing all the informed answers (“1”, “0”, or “0.5”) and dividing this sum by the number of informed answers. We left the blanks out of the calculation in both the numerator and denominator. Ranging from zero to 100%, the intermediate RPS measured the collective optimism of the scorers and informed experts on the likelihood that the research question would satisfy a specific criterion. This method of dealing with missing answers/blanks increases the accuracy of collective prediction by allowing experts who do not necessarily know to adequately score a research question against each criterion to withdraw from answering [33]. We calculated the unweighted RPS as the mean of all five intermediate priority scores.

In the next step, we calculated weights by dividing the observed average rank for each criterion by the expected average rank where all five criteria are equally important (which should be 3.00) [34]. In our exercise, for each scored research question, the intermediate score for effectiveness and impact on burden criteria were increased by 8%, there was almost no change in score for the answerability criteria, and the score decreased by 7% and 10% for deliverability and equity criteria, respectively (**Table 2**). We multiplied the weights with the intermediate scores of each criterion to calculate weighted intermediate scores and computed the weighted RPS as the mean of all the weighted intermediate scores.

We also calculated the average expert agreement (AEA) for each of the 95 research questions using the formula below [26]. The AEA informed us about the proportion of scorers who gave the same most frequent response for all the informed answers (“1”, “0”, or “0.5”) [35,36]. It is a measure of concurrence/disagreement in the scorers’ opinion around the RPS and is unaffected by the varying number and composition of scorers per criterion [37].



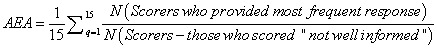



### Ethics

We obtained ethical approval for the study from the research and ethics review committees of International Centre for Diarrhoeal Disease Research Bangladesh (icddr,b) (PR-18055). Experts provided informed consent during the start of both online surveys, and participants were aware that they could exit the surveys at any time without any penalty. We maintained the participants’ privacy and confidentiality during data collection, management, and analysis, and used contact information only to send survey invitations and reminders. We removed personal identifiers (i.e. names) before analysis and kept the data in secure and password-protected devices.

## RESULTS

### Research priorities for LMCIs

The overall weighted RPS for the 95 research questions ranged from 0.903 (highest) to 0.638 (lowest). The AEA ranged from 0.85 to 0.66, and we observed a positive association between AEA and RPS (Table S1 in the **Online Supplementary Document**).

The top-ranked research priority was identifying barriers and challenges to implementing facility-based KMC in LMICs (#1). Among the top 20 research priorities for LMICs (**Table 4**), six were focused on various aspects of KMC, including assessing the effectiveness of community-based KMC (#6), increasing acceptability and compliance of KMC (#8), determining clinical outcomes of preterm newborns discharged to continue KMC at home (#10), and assessing the impact of quality improvement initiatives in improving KMC counselling (#14). The remainder of the top 20 research priorities focused on promoting early and exclusive breastfeeding (#4), health systems-related research such as assessing the impact of the availability of skilled birth and neonatal care attendants on survival outcomes of babies (#2), strengthening referral systems for women and preterm/LBW newborns (#3 and #5), evaluating quality improvement activities (#17), exploring barriers and facilitators to using antenatal steroid use in health facilities (#19) and post-discharge follow-up of preterm newborns (#12 and #20). Several of the top 20 questions also focused on screening of high-risk or general population women (#11), understanding the cause or managing preterm babies with illnesses including jaundice, sepsis and retinopathy of prematurity (#7, #13, #15, and #16).

**Table 4:** Top 20 research questions for LMICs (n = 109) with scores for each criterion, overall weighted RPS, and AEA

Rank	Research questions	Domain	Answerability intermediate RPS	Effectiveness intermediate RPS	Deliverability intermediate RPS	Burden intermediate RPS	Equity intermediate RPS	Weighted RPS	AEA
1	What are the barriers and challenges of implementing facility-based KMC in LMICs?	HPSR	0.984	0.952	0.945	0.747	0.895	0.903	0.847
2	How does the presence of skilled birth and neonatal care attendants influence survival outcomes for babies?	HPSR	0.928	0.944	0.923	0.737	0.918	0.888	0.823
3	How can referral network and systems be strengthened for timely referral of women experiencing obstetric emergencies (including preterm labour) from rural/primary care to higher care level facilities in LMICs?	HPSR	0.923	0.934	0.884	0.712	0.959	0.880	0.809
4	How to promote early initiation and exclusive breast feeding of preterm, LBW and SGA infants in LMICs?	RIEI	0.916	0.943	0.929	0.706	0.871	0.871	0.800
5	How can transport and referral systems for preterm, LBW, SGA newborns be improved or maximised in LMICs?	HPSR	0.936	0.912	0.877	0.721	0.920	0.871	0.813
6	Assess the effectiveness of community-based KMC in reducing neonatal mortality of clinically stable preterm and LBW infants?	HPSR	0.944	0.929	0.907	0.698	0.879	0.870	0.810
7	What is the prevalence and cause of neonatal sepsis in preterm, LBW and SGA infants in LMICs?	EPI	0.952	0.897	0.895	0.720	0.876	0.866	0.787
8	How can acceptability and compliance of KMC be increased in LMICs?	RIEI	0.911	0.926	0.921	0.720	0.851	0.865	0.789
9	What are barriers and challenges to improving existing skin-to-skin practice in LMICs?	HPSR	0.961	0.928	0.938	0.662	0.819	0.860	0.807
10	What are the clinical outcomes of preterm newborns discharged to continue KMC at home?	EPI	0.950	0.897	0.920	0.653	0.880	0.857	0.798
11	Evaluate interventions to screen women at risk of PTB during ANC (e.g. anaemia, preeclampsia, NCDs, malnutrition) and improve maternal and newborn outcomes in LMICs.	HPSR	0.927	0.893	0.894	0.660	0.877	0.847	0.769
12	Develop effective strategies to improve post discharge follow-up of preterm, LBW and SGA infants in LMICs.	RDNI	0.910	0.885	0.865	0.677	0.878	0.841	0.756
13	What are barriers of doing ROP screening for all eligible preterm babies in LMICs?	HPSR	0.966	0.907	0.838	0.686	0.803	0.840	0.761
14	Assess the impact of quality improvement initiatives in improving KMC counselling.	HPSR	0.905	0.912	0.920	0.634	0.839	0.839	0.779
15	How can we provide safe and effective phototherapy for premature neonates in LMICs?	RIEI	0.922	0.912	0.861	0.655	0.855	0.839	0.777
16	What intervention packages can be developed to manage premature and small infants with neonatal jaundice in LMICs?	RDNI	0.894	0.898	0.889	0.701	0.809	0.837	0.771
17	Evaluate the effectiveness and cost-effectiveness of QI activities in improving care of preterm babies at health facilities in LMICs.	HPSR	0.886	0.893	0.865	0.695	0.847	0.836	0.746
18	What is the effect of nutritional status (e.g. underweight, overweight and obesity, micronutrient deficiency etc.) on LBW, SGA and PTB in LMICs?	EPI	0.931	0.868	0.885	0.671	0.832	0.835	0.755
19	Explore barriers and facilitators to antenatal steroid use in public health facilities in LMICs.	HPSR	0.921	0.898	0.902	0.669	0.782	0.833	0.766
20	Evaluate the use of digital technologies (e.g. mobile phone etc.) to improve follow-up of preterm babies after discharge from health facilities.	RIEI	0.935	0.876	0.872	0.646	0.838	0.831	0.757

LMICs – low and middle-income countries, KMC - Kangaroo Mother Care, RPS – research priority scores, AEA – average expert agreement, HPSR – health policy and systems research, EPI – epidemiological research, ANC – anteanatal care, RIEI – research to improve existing interventions, RDNI – research to develop new interventions, LBW – low birth weight, SGA – small for gestational age, PTB – preterm birth, ROP – retinopathy of prematurity, QI – quality improvement

Eleven (55%) of the top 20 research priorities were categorised as health systems and policy research, four as research to develop existing interventions (20%), three as epidemiological research, and two as the development of new interventions.

#### Top-ranked priorities across research criteria in LMICs

The research question on identifying barriers and challenges of implementing facility-based KMC received the highest score for four criteria – the likelihood of burden reduction, answerability, effectiveness, and deliverability **(**Table S2 in the **Online Supplementary Document****)**. For the criteria reduction of the burden, the other two leading research questions that experts agreed on were determining the impact of skilled birth and neonatal care attendants on survival outcomes for babies and improving transport and referral systems for preterm, LBW, or small for gestational age (SGA) infants in LMICs. There was also high agreement that improving or strengthening referral and transport systems for women and newborns, followed by assessing the impact of the presence of skilled birth and neonatal care attendants on survival outcomes for babies, would improve equity (Table S2 in the **Online Supplementary Document**).

### Subgroup analyses

We conducted subgroup analyses for Bangladesh-based (**Table 5**) and international scorers (**Table 6**) outside Bangladesh to search for any variations in priorities identified. We also compared the ranks for each research priority between these groups and the overall LMIC scores. Ranks within the subgroups over a 10-point deviation compared to the LMIC ranks are marked with an asterisk (*) in **Table 7**.

**Table 5:** Top 20 research questions for Bangladesh (n = 54) with scores for each criterion, overall weighted RPS, and AEA

Rank	Research questions	Domain	Answerability intermediate RPS	Effectiveness intermediate RPS	Deliverability intermediate RPS	Burden intermediate RPS	Equity intermediate RPS	Weighted RPS	AEA
1	How does the presence of skilled birth and neonatal care attendants influence survival outcomes for babies?	HPSR	0.956	0.965	0.948	0.752	0.947	0.911	0.860
2	What are the barriers and challenges of implementing facility-based KMC in LMICs?	HPSR	0.982	0.960	0.942	0.752	0.919	0.909	0.859
3	What is the prevalence and cause of neonatal sepsis in preterm, LBW and SGA infants in LMICs?	EPI	0.969	0.940	0.927	0.773	0.897	0.900	0.844
4	How can referral network and systems be strengthened for timely referral of women experiencing obstetric emergencies (including preterm labour) from rural/primary care to higher care level facilities in LMICs?	HPSR	0.944	0.954	0.935	0.718	0.965	0.900	0.840
5	How can acceptability and compliance KMC be increased in LMICs?	RIEI	0.924	0.956	0.952	0.750	0.864	0.888	0.827
6	How to promote early initiation and exclusive breast feeding of preterm, LBW and SGA infants in LMICs?	RIEI	0.941	0.954	0.943	0.708	0.904	0.887	0.821
7	Assess the effectiveness of community-based KMC in reducing neonatal mortality of clinically stable preterm and LBW infants?	HPSR	0.972	0.937	0.919	0.711	0.901	0.886	0.834
8	What are the clinical outcomes of preterm newborns discharged to continue KMC at home?	EPI	0.954	0.938	0.938	0.681	0.900	0.880	0.825
9	Assess the impact of quality improvement initiatives in improving KMC counselling.	HPSR	0.946	0.963	0.955	0.663	0.884	0.879	0.830
10	What are barriers and challenges to improving existing skin-to-skin practice in LMICs?	HPSR	0.955	0.944	0.934	0.712	0.857	0.879	0.844
11	How can transport and referral systems for preterm LBW, SGA newborns be improved or maximised in LMICs?	HPSR	0.939	0.914	0.923	0.722	0.909	0.879	0.837
12	What is the effect of antepartum complications in the current pregnancy (e.g. multiple gestation, cervical incompetence, preeclampsia, eclampsia, hypertension, diabetes etc.) on LBW, SGA and PTB in LMICs?	EPI	0.972	0.950	0.912	0.735	0.792	0.873	0.803
13	What is the effect of nutritional status (e.g. underweight, overweight and obesity, micronutrient deficiency etc.) on LBW, SGA and PTB in LMICs?	EPI	0.945	0.927	0.951	0.719	0.827	0.873	0.817
14	Evaluate interventions to screen women at risk of PTB during ANC (e.g. anaemia, preeclampsia, NCDs, malnutrition) and improve maternal and newborn outcomes in LMICs.	HPSR	0.941	0.916	0.914	0.700	0.903	0.872	0.805
15	What are barriers of doing ROP screening for all eligible preterm babies in LMICs?	HPSR	0.965	0.915	0.896	0.729	0.841	0.868	0.808
16	How can care for preterm and LBW newborns in remote community settings be improved?	HPSR	0.917	0.935	0.905	0.687	0.901	0.866	0.794
17	What are the short- and long-term health and developmental outcomes of babies born preterm, LBW, or SGA in LMICs?	EPI	0.951	0.926	0.933	0.673	0.847	0.864	0.787
18	How can clinical support and supervision of community health workers in the management of small and sick newborns be improved in LMICs?	HPSR	0.945	0.891	0.913	0.692	0.884	0.862	0.787
19	What are the barriers and enablers of improved accuracy of gestational age assessment in LMICs?	HPSR	0.946	0.936	0.886	0.685	0.851	0.860	0.803
20	Evaluate the effectiveness and cost-effectiveness of nutritional interventions in improving nutritional status of preterm infants in LMICs.	EPI	0.937	0.893	0.892	0.692	0.899	0.860	0.807

LMICs – low and middle-income countries, KMC - Kangaroo Mother Care, RPS – research priority scores, AEA – average expert agreement, HPSR – health policy and systems research, EPI – epidemiological research, RIEI – research to improve existing interventions, RDNI – research to develop new interventions, LBW – low birth weight, SGA – small for gestational age, PTB – preterm birth, ROP – retinopathy of prematurity, NCD – non-communicable disease

**Table 6:** Top 20 research questions scored by international experts (n = 55) with scores for each criterion, overall weighted RPS, and AEA

Rank	Research questions	Domain	Answerability intermediate RPS	Effectiveness intermediate RPS	Deliverability intermediate RPS	Burden intermediate RPS	Equity intermediate RPS	Weighted RPS	AEA
1	What are the barriers and challenges of implementing facility-based KMC in LMICs?	HPSR	0.987	0.938	0.949	0.739	0.856	0.893	0.826
2	How can transport and referral systems for preterm, LBW, SGA newborns be improved or maximised in LMICs?	HPSR	0.931	0.908	0.787	0.719	0.938	0.855	0.771
3	Determine the effectiveness and cost-effectiveness of various strategies (e.g. CPAP, high flow oxygen, T-piece resuscitation etc.) for treating preterm infants with respiratory failure in health facilities in LMICs.	RIEI	0.867	0.955	0.845	0.682	0.893	0.847	0.777
4	How does the presence of skilled birth and neonatal care attendants influence survival outcomes for babies?	HPSR	0.872	0.903	0.873	0.711	0.867	0.844	0.751
5	How can referral network and systems be strengthened for timely referral of women experiencing obstetric emergencies (including preterm labour) from rural/primary care to higher care level facilities in LMICs?	HPSR	0.883	0.897	0.791	0.703	0.950	0.843	0.753
6	Assess the effectiveness of community-based KMC in reducing neonatal mortality of clinically stable preterm and LBW infants?	HPSR	0.889	0.913	0.882	0.669	0.833	0.836	0.759
7	How to promote early initiation and exclusive breast feeding of preterm, LBW and SGA infants in LMICs?	RIEI	0.862	0.918	0.897	0.700	0.802	0.835	0.757
8	Explore barriers and facilitators to antenatal steroid use in public health facilities in LMICs.	HPSR	0.928	0.878	0.921	0.683	0.731	0.828	0.741
9	What are barriers and challenges to improving existing skin-to-skin practice in LMICs?	HPSR	0.970	0.899	0.944	0.571	0.756	0.826	0.762
10	Develop effective strategies to improve post discharge follow-up of preterm, LBW, and SGA infants in LMICs.	RDNI	0.914	0.853	0.814	0.652	0.895	0.823	0.721
11	How can acceptability and compliance of KMC be increased in LMICs?	RIEI	0.884	0.866	0.858	0.658	0.829	0.817	0.712
12	Understand the epidemiology of nosocomial infections in newborn nurseries or SCANU in LMICs.	EPI	0.915	0.873	0.902	0.658	0.692	0.808	0.731
13	What intervention packages can be developed to manage premature and small infants with neonatal jaundice in LMICs?	RDNI	0.891	0.892	0.850	0.660	0.742	0.807	0.713
14	What are the clinical outcomes of preterm newborns discharged to continue KMC at home?	EPI	0.942	0.806	0.881	0.589	0.838	0.807	0.739
15	Evaluate the use of digital technologies (e.g. mobile phone etc.) to improve follow-up of preterm babies after discharge from health facilities.	RIEI	0.932	0.810	0.854	0.617	0.819	0.803	0.720
16	Can providing proper training to community health workers ensure community continuation of KMC through domiciliary follow-up?	RIEI	0.941	0.879	0.843	0.519	0.851	0.802	0.759
17	What is the prevalence and cause of neonatal sepsis in preterm, LBW and small for gestational age SGA infants in LMICs?	EPI	0.922	0.818	0.838	0.611	0.840	0.802	0.705
18	Evaluate interventions to screen women at risk of PTB during ANC (e.g. anaemia, preeclampsia, NCDs, malnutrition) and improve maternal and newborn outcomes in LMICs.	HPSR	0.899	0.845	0.854	0.586	0.831	0.799	0.703
19	Assess the effect of ECD interventions (e.g. early infant stimulation/parenting interventions) on health and developmental outcomes of preterm newborn.	EPI	0.899	0.873	0.796	0.603	0.813	0.795	0.717
20	Evaluate the effectiveness and cost-effectiveness of QI activities in improving care of preterm babies at health facilities in LMICs.	HPSR	0.875	0.803	0.795	0.670	0.828	0.792	0.664

SCANU – specialised care newborn units, LMICs – low and middle-income countries, KMC - Kangaroo Mother Care, RPS – research priority scores, AEA – average expert agreement, HPSR – health policy and systems research, ANC – antenatal care, CPAP – continuous positive airway pressure, EPI – epidemiological research, RIEI – research to improve existing interventions, RDNI – research to develop new interventions, LBW – low birth weight, SGA – small for gestational age, PTB – preterm birth, ROP – retinopathy of prematurity, QI – quality improvement

**Table 7:** Comparison of ranks within subgroup analyses (geographical location)

Rank	Research questions	LMIC, total n = 109	Bangladesh participants (n = 54)	International participants (n = 55)	International HIC (n = 32)	International LMIC (n = 23)
1	What are the barriers and challenges of implementing facility-based KMC in LMICs?	1	2	1	1	1
2	How does the presence of skilled birth and neonatal care attendants influence survival outcomes for babies?	2	1	4	5	12*
3	How can referral network and systems be strengthened for timely referral of women experiencing obstetric emergencies (including preterm labour) from rural/primary care to higher care level facilities in LMICs?	3	4	5	6	11
4	How to promote early initiation and exclusive breast feeding of preterm, LBW and SGA infants in LMICs?	4	6	7	2	24*
5	How can transport and referral systems for preterm, LBW, SGA newborns be improved or maximised in LMICs?	5	11	2	3	7
6	Assess the effectiveness of community-based KMC in reducing neonatal mortality of clinically stable preterm and LBW infants?	6	7	6	13	3
7	What is the prevalence and cause of neonatal sepsis in preterm, LBW and SGA infants in LMICs?	7	3	17*	29*	6
8	How can acceptability and compliance of KMC be increased in LMICs?	8	5	11	10	17
9	What are barriers and challenges to improving existing skin-to-skin practice in LMICs?	9	10	9	9	8
10	What are the clinical outcomes of preterm newborns discharged to continue KMC at home?	10	8	14	12	20*
11	Evaluate interventions to screen women at risk of PTB during ANC (e.g. anaemia, preeclampsia, NCDs, malnutrition) and improve maternal and newborn outcomes in LMICs.	11	14	18	17	19
12	Develop effective strategies to improve post discharge follow-up of preterm, LBW, and SGA infants in LMICs.	12	26*	10	7	18
13	What are barriers of doing ROP screening for all eligible preterm babies in LMICs?	13	15	23*	15	36*
14	Assess the impact of quality improvement initiatives in improving KMC counselling.	14	9	29*	43*	27*
15	How can we provide safe and effective phototherapy for premature neonates in LMICs?	15	24	21	24	9
16	What intervention packages can be developed to manage premature and small infants with neonatal jaundice in LMICs?	16	27*	13	22	16
17	Evaluate the effectiveness and cost-effectiveness of QI activities in improving care of preterm babies at health facilities in LMICs.	17	22	20	28*	15
18	What is the effect of nutritional status (e.g. underweight, overweight and obesity, micronutrient deficiency etc.) on LBW, SGA, and PTB in LMICs?	18	13	25	14	37*
19	Explore barriers and facilitators to antenatal steroid use in public health facilities in LMICs.	19	36*	8*	8*	14
20	Evaluate the use of digital technologies (e.g. mobile phone etc.) to improve follow-up of preterm babies after discharge from health facilities.	20	29	15	36*	4*

LMICs – low and middle-income countries, KMC - Kangaroo Mother Care, RPS – research priority scores, LBW – low birth weight, SGA – small for gestational age, PTB – preterm birth, ROP – retinopathy of prematurity, QI – quality improvement, HIC – high-income country, ANC – antenatal care

*Cells with ranks over a 10-point deviation from the LMIC ranks.

#### Research priorities for Bangladesh

The RPS ranged from 0.911 to 0.719, and the AEA ranged from 0.82 to 0.58. There was considerable overlap between research priorities in LMICs and Bangladesh, with 16 questions of the top 20 questions in Bangladesh appearing in the overall LMIC list (**Table 7** and Table S3 in the **Online Supplementary Document**). The top research priority was assessing the impact of the availability of skilled birth and neonatal care attendants on the survival outcomes of babies (**Table 7**). Like that of LMICs, six of the 20 Bangladesh-based research questions were on KMC (#2, #5, #7, #8, #9, and #10), while others included understanding the burden and cause of sepsis in preterm newborns (#3), strengthening referral systems (#4 and #11), promoting early and exclusive breastfeeding (#6), screening of high-risk women (#14), and exploring barriers of screening children with retinopathy of prematurity (#15). Experts also gave high scores to several epidemiological questions focusing on short and long-term developmental outcomes in preterm newborns, the accuracy of gestational age estimation, and understanding the effects of antepartum complications, nutritional status, and nutritional interventions on preterm birth outcomes. Two questions also specifically focused on improving care at the remote community level (#16) and improving support and supervision of community health workers (#18).

#### Research priorities identified by global/international scorers

We also observed a high overlap (16 questions) between the top 20 questions identified by LMIC and international experts **(****Table 5** and **Table 6****)**. The top-ranked research priority proposed to identify barriers and challenges to implementing facility-based kangaroo mother care in LMICs (#1) (**Table 5**). International scorers also highlighted questions such as determining the effectiveness and cost-effectiveness of various strategies (e.g. continuous positive airway pressure (CPAP), high flow oxygen, T-piece resuscitation, etc.)) for treating preterm infants (#3), understanding the epidemiology of nosocomial infections in newborn units (#12), improving community continuation of KMC by proper training to community health workers (#16), and assessing the effect of early childhood development interventions in improving preterm newborn (#19) outcomes as important (**Table 5**). We further stratified the analysis by international scorers based on high-income countries (HICs) and LMICs (Table S5 and S6 in the **Online Supplementary Document****)**; 14 of the top 20 questions prioritised by researchers in HICs and 16 prioritised by those in LMICs appeared in the overall LMIC list **(****Table 7** and Table S7 in the **Online Supplementary Document****)**.

#### Research priorities segregated by the expertise of scorers

We also attempted to classify research priorities by the expertise of scorers listed in **Table 3**. While we observed some differences among individuals with expertise in laboratory science research (LSR), we found an overlap in about 15-18 questions in the top 20 between the different groups and the overall LMIC list. We did observe a substantial difference among those experts in LSR and the overall LMIC list (Tables S8-13 in the **Online Supplementary Document**).

## DISCUSSION

Many children are still being born preterm and are suffering from short- and long-term consequences of preterm-related complications. With only a decade left to reach the SDGs, many countries will fail to achieve the targets related to child mortality and newborn mortality unless the challenges in the prevention and management of preterm birth are addressed urgently [2,4,5]. As such, this priority-setting exercise aimed to identify research questions that reflect the knowledge gaps that need to be addressed to accelerate progress in this area in LMICs and Bangladesh within the SDG era. Overall, participating experts have prioritised research questions primarily aimed at improving the survival of preterm infants rather than identifying long-term potential preventative solutions. The survey results have strongly prioritised health policy and systems research to understand barriers and improve effectiveness, deliverability, acceptance, and uptake of evidence-based interventions combined with other epidemiological research to address the critical gaps in knowledge in resource-poor settings. This is in line with the distribution in other CHNRI exercises, where health policy and systems-related research questions were more prioritised than other research types primarily for their ability to immediately address the disease burden in low-resource settings [31]. The positive association between expert agreement and research priority scores also indicates substantial agreement in the high ranked priorities among experts.

Of the top 20 research questions in LMICs or Bangladesh, six were related to identifying gaps or challenges or improving the implementation of either facility-based or community-based KMC. Although KMC has been identified and epitomised as a critical intervention in reducing mortality and morbidity in preterm infants (including ENAP), as recommended by the World Health Organization (WHO), its application and scale-up, especially in LMIC settings, have been challenging due to health systems bottlenecks and demand side barriers such as poor quality, lack of awareness, acceptability, and access [38-40]. For example, the Government of Bangladesh adopted KMC as the primary approach for averting preterm-related deaths through the Promise Renewed Declaration in 2013 [41]. Despite this, to date, KMC has been scaled to only around 400 facilities in Bangladesh, and only 12 896 of the estimated 573 000 preterms and 192 000 low-birth-weight babies (<5%coverage) received KMC services at health facilities in 2022 [42]. Consequently, this exercise has identified relevant research priorities that aim to identify gaps in the implementation/scale-up of facility-based KMC, improve the quality of KMC counselling, and improve acceptability and compliance with KMC. Although a few studies have been launched recently [43-47], there is still limited evidence on the benefits of community KMC (cKMC) or community continuation of facility-based KMC, and area that has also been prioritised in this exercise. Research priority-setting exercises during [32,33] and after the MDG era [37,48,49] have also highlighted similar research questions confirming that stark evidence gaps in implementing KMC services remain. For example, Yoshida et al. [37], Alobo et al. [48], and Souza et al. [49] conducted CHNRI exercises to determine research priorities for maternal and newborn health the post-MDG area in the global and African contexts. Research on KMC was featured in the top-10 priorities in those exercises and included evaluation of the impact of cKMC on neonatal mortality, improving utilisation of KMC at the community level, evaluating coverage, identifying facilitators and barriers, and scaling up of facility based KMC [37,48,49].

Despite increases in access to institutional care, about one-third of women in LMICs still deliver at home without adequately skilled staff or cannot timely access care due to poor referral and transport mechanisms linking women and newborns to care [42,50,51]. Similarly, a substantial proportion of newborn deaths in LMICs still occur at home, with delays and challenges in accessing care. There is, therefore, a need to implement interventions across the continuum of care and in the community, improving maternal and newborn survival by helping families to adopt sound health practices, identifying high-risk women, encouraging facility delivery, appropriate care-seeking and ensuring timeous referral for mothers at risk of preterm birth and sick preterm newborns. To date, there is little implementation research on establishing a responsive and equitable referral mechanism to facilitate the transfer of women and newborns from home to facilities or between facilities during the small and often fatal window of time around delivery [51-54]. Additionally, due to the insufficient availability and distribution of neonatal intensive care or specialised newborn units in low-resource settings, reliable and well-equipped transportation is often a challenging and neglected missing link to timely emergency care, with systematic reviews indicating scarcity of high-level evidence relating to effective implementation of neonatal transport in developing countries [55-57]. Also of concern is the gap in the quality of inpatient services provided to women and newborns, both in terms of actual facility capability or readiness and provider’s knowledge and competencies, with many studies indicating services that are delayed, inadequate, unnecessary, harmful, and disrespectful, and that result in easily avoidable deaths [58-65]. It is, therefore, fitting that the top research priorities identified in this study relate to understanding the impact of skilled birth and neonatal care attendance on newborn outcomes, strengthening referral and transport linkages for critically sick mothers and preterm newborns, evaluating approaches to identifying pregnancies at most significant risk of preterm birth, and improving quality of care for sick preterm newborns at health facilities. The participating experts also emphasised the need for studies to improve the care of preterm newborns at the community level and follow-up of preterm newborns once discharged from facilities. Other global or regional CHNRI priority-setting exercises on maternal and newborn health have also highlighted similar research questions related to breastfeeding [37,66], referral and transport [48,49], improvement in the quality of maternal and newborn care at facilities [37,48], and community-based care [37,49,66].

This priority-setting exercise has also highlighted several epidemiological studies, mainly from experts from Bangladesh, szch as determining the prevalence and cause of sepsis in preterm newborns, understanding the effect of antepartum complications, nutritional status or nutritional interventions ECD interventions on birth outcomes or preterm babies. This indicates that, despite evidence from many HICs, there is still a lack of evidence regarding epidemiological studies from many LMICs including Bangladesh [67].

Although most participating experts had experience conducting research in LMICs, we attempted to stratify our analysis based on their geographical location and by expertise of scorers. While our findings indicate a high overlap in the top 20 research priorities, there is a 10%-30% divergence between groups. For example, while international experts have prioritised research related to the implementation of antenatal corticosteroids, determining the effectiveness and cost-effectiveness of various strategies (e.g. CPAP, high flow oxygen, T-piece resuscitation, etc.) for treating preterm infants, the effect of ECD interventions on preterm newborns, epidemiology of nosocomial infections in newborn nurseries or SCANU, experts from Bangladesh ranked them as #36, #49, #48, and #52, respectively; they also focused on questions related to improving care for newborns at the community level in the top 20, which were given lower scored by international participants. Similarly, differences were observed by scorer’s expertise, especially those with expertise in LSR, possibly due to the low response in this category (n = 8). Previous exercises have highlighted similar discordance between regional, international, or high and LMIC contexts or by expertise [36,48,68,69]. Despite a general agreement among the key research priorities, this disagreement may be due to differences in the groups’ characteristics and and differential requirements in the different contexts [36,48,69]. A scoping review of systematic reviews found a lack of primary research evidence on developing and testing interventions for the prevention of spontaneous preterm birth from low-income country settings and emphasised that this may lead to a risk of inappropriate and unsafe recommendations for practice within those contexts. It is, therefore, essential to highlight and consider this disagreement to ensure that funding allocation decisions and proposals for future work are in line with the contextual needs.

Analysis of research questions by criterion revealed how the criteria could be used to determine the strengths and weaknesses of specific research questions [68]. For example, strengthening referral and transport systems for mothers experiencing obstetric emergencies was ranked the #3 research priority in LMICs and received the highest scores in the “equity” criteria. However, it ranked #18 according to the “answerability” criteria, indicating a potential for difficulty in designing and implementing a study to appropriately address this issue.

One of this exercise's main strengths is adapting the CHNRI methodology. Apart from being transparent, structured, and flexible, other practical benefits are its low cost and the ease of conducting it online. However, we also faced some challenges. Our survey response rates were low during the elicitation of research questions and the scoring process. This is not uncommon with this method [31,70] and may result in a self-selection bias. Yoshida et al. [71], however, found that the collective opinion of an expert group in ranking research questions using categorical variables (yes/no/not sure/do not know) stabilises quickly, resulting in a high degree of reproducibility of the top 15-20 ranked research questions with only 45-55 experts [71]. To improve response rates and ensure that a diverse group of experts participated, we used structured and unstructured methods to list a large pool of experts and sent them regular requests and reminders to participate in the study. We also invited the larger pool of experts (and not only those who submitted research questions) to participate in the scoring process [31], which increased the number of people participating and allowed individuals who were hesitant to provide research questions or may not have had to do so another opportunity to contribute [70]. About half of the experts who participated in the scoring process were from Bangladesh, which enabled us to conduct a country-level analysis and present the findings to key stakeholders at the national level in Bangladesh.

The list of research questions to be scored was also long and time-consuming to collect, resulting in scorer fatigue. For the set of 95 research questions, each respondent had to provide a total of 1425 (95 × 15) scores, which took over an hour to complete. About 61% of the scorers had completed the scoring process in full, and about 69% scored at least half of the questions. To reduce preferential bias due to scoring fatigue, we randomly presented research questions to each scorer, ensuring that all questions had an equal chance of being scored [48,72] and allowing us to include responses from experts who had scored at least one full question.

Other potential biases inherent in this methodology include the possibility of excluding valuable research ideas during the research question elicitation phase or consolidation phase [31]. While the range of research ideas is infinite, through this process, we obtained a good coverage of ideas related to preventing and managing preterm birth across the four research domains. Additionally, more than 90% of the experts participating in the surveys had experience working in an LMIC setting or Bangladesh, which enhanced the chances of receiving contextually relevant ideas. George et al. [33] suggest that scores for missing research questions could be estimated by relating them to a similar question or having it scored by one or a group of experts and then comparing the scores.

Engaging a diverse group of stakeholders has improved the legitimacy, credibility, inclusiveness, contextual relevance, and ownership of the prioritised research, leading to greater investment opportunities [34,73-75]. However, identifying and engaging stakeholders per the original CHNRI method has been challenging [75]. Following previous exercises [35,76], we asked our technical experts to rank the five pre-selected criteria and participate in the idea generation and scoring processes. They assigned higher scores to “answerability”, and “effectiveness” and lowest scores to the “burden reduction” criterion, yet they allocated a greater weight to “effectiveness” and “burden reduction” compared to “answerability” or “delivery”. Consequently, we observed changes in ranks between weighted and unweighted scores. However, the top 20 questions remained almost identical [75]. Assignment of domains to research priorities was also prone to subjective variation in the interpretation of domains [36]. To reduce bias, the two primary authors independently assigned domains to the research priorities, and a senior researcher resolved any disagreements.

This exercise, drew on the expertise of a diverse group of participants who contributed to and scored questions to develop a set of research priorities that reflect the needs in Bangladesh and other LMICs on prevention and management of preterm birth going forward. The findings provide guidance to national level and LMICs stakeholders on future research investments in the area. Prior reviews of research priority setting exercises have continually stressed the importance of having a dissemination strategy with key stakeholders for optimal uptake of the identified priorities [77]. As such, dissemination will be done with relevant experts and stakeholders including policy makers and programme implementers from the Ministry of Health and Family Welfare, researchers, development partners and donors in Bangladesh. This should be coupled with regular and continuous monitoring of research investments and progress in key standardised outcome indicators related to prevention and management of preterm birth.

## CONCLUSIONS

Preterm birth is the leading cause of newborn deaths in LMICs. Achieving the highly ambitious SDG target of reducing neonatal mortality rate (NMR) to ≤12 per 1000 live births will require accelerated efforts to prevent and manage preterm birth and its complications. The findings from this study offer a set of prioritised research questions related to improving the prevention of preterm birth and care and management of the preterm baby, which, we hope, will help bring further attention and more secure funding from donors, researchers and policymakers globally, in LMICs and Bangladesh.

## Additional material


Online Supplementary Document
